# Core Loss Modeling of Magnetic Components Using a Data-Driven Method

**DOI:** 10.3390/s26102919

**Published:** 2026-05-07

**Authors:** Xinjian Gao, Shizhuang Yin, Zhonghua Cheng, Tielu Gao, Enzhi Dong

**Affiliations:** Shijiazhuang Campus, Army Engineering University of PLA, Shijiazhuang 050003, China; 13912453267@163.com (X.G.); yinshizhuang111@sina.com (S.Y.); hello202511@yeah.net (E.D.)

**Keywords:** eXtreme gradient boosting, simulated annealing algorithms, confusion matrices, mean squared error, mean absolute error, magnetic core loss

## Abstract

Research on loss characteristics of magnetic components is a critical topic in power conversion technology. To address the significant discrepancies between existing core loss models and practical application requirements, this paper establishes a high-precision core loss evaluation model based on eXtreme Gradient Boosting. The model uses temperature, material, and excitation waveform as decision variables, with minimizing core loss as the objective. The study first evaluates the model through confusion matrices while conducting single-factor analyses of temperature, excitation waveform, and material using boxplots and investigating synergistic interactions between single factors. Subsequently, key metrics including coefficient of determination, mean squared error, and mean absolute error are employed for comprehensive regression assessment. Residual plots are utilized to examine the fit between predicted and actual values, demonstrating the model’s high accuracy, strong applicability, and practical relevance, thereby validating its superiority. Finally, the paper explores maximizing magnetic energy transmission by solving the optimization problem using simulated annealing algorithms to determine conditions that achieve both minimum core loss and maximum transmission efficiency, extending the research to multi-objective optimization.

## 1. Introduction

In recent years, the development of electric energy conversion technology has been rapid, especially in the fields of new energy and information communication, which are widely used [1]. Electricity conversion refers to the conversion of electrical energy from one form to another (such as direct current of different voltages, alternating current of different frequencies and sizes, etc.) [2]. Due to the development of third-generation power semiconductor technology, high frequency, high power density, and high reliability have become the development direction of power converter products [3]. Magnetic components (transformers, inductors, etc.) are essential components in power converters, responsible for the transmission, storage, filtering, and other functions of magnetic energy. They have a significant impact on the volume, weight, losses, and cost of power converters [4]. In order to achieve high efficiency and high power density design, in addition to meeting the feasibility design of magnetic component electrical parameters, they are also required to have low losses [5]. Therefore, it is necessary to conduct a detailed study and analysis of the loss characteristics of magnetic components, which include winding losses and core losses [6]. The winding loss of copper conductors can be accurately obtained through electromagnetic field finite element simulation technology, but the core loss is the power loss generated by magnetic materials under high-frequency alternating magnetic flux [7]. Due to the complex production processes and stringent manufacturing technologies required for electrical steel materials (such as iron cores for motors and transformers), their losses are related to various factors such as excitation waveform, operating temperature, magnetic core material, operating frequency, and magnetic flux density [8]. There is a significant difference between the existing loss models of magnetic core materials and the actual application requirements.

**Figure 1:**
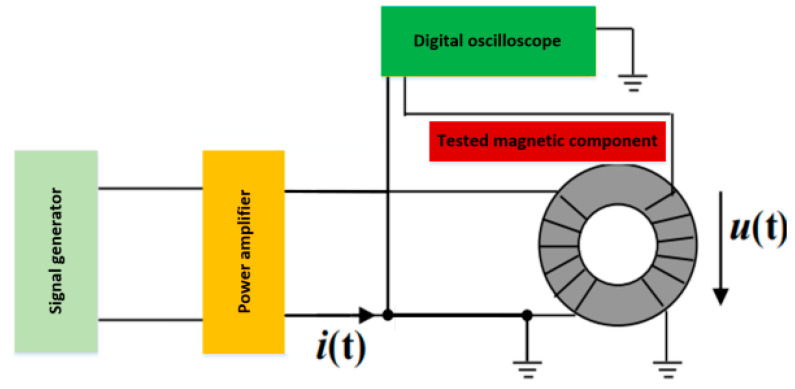
Measurement of magnetic core loss using dual winding method.

The measurement of magnetic core loss currently generally adopts the AC power method [9]. As shown in Figure 1, the tested magnetic core generally adopts a circular ring shape (the average magnetic circuit length is $l_{e}$; the cross-sectional area of the magnetic core is $A_{e}$), and the excitation winding and induction winding are uniformly wound on the magnetic core ($N_{1}$ and $N_{2}$ are the turns of the excitation winding and induction winding, respectively, generally taken as $N_{1}$ = $N_{2}$). The signal generator generates a sine wave or other waveform of a given frequency f (period is T=1/f), which is applied to the excitation winding as a high-frequency excitation source through a high-frequency power amplifier. According to Ampere’s Loop Law, the excitation current i(t) of the winding generates a magnetic field strength H(t) on the magnetic core (magnetic field strength refers to the Lorentz force experienced by a unit current element in the magnetic field, which is an important parameter for describing the strength of the magnetic field). The magnetic field strength acts on the magnetic core of the conductive material to generate magnetic flux density B(t) (magnetic flux density refers to the magnetic flux perpendicular to the magnetic field lines per unit area, that is, the magnetic flux perpendicular to the direction of the magnetic field per unit area) [10]. According to the law of electromagnetic induction, the alternating magnetic flux in the magnetic core generates an induced voltage u(t) on the induction winding.

Further, by collecting the excitation current i(t) of the tested magnetic core winding, the magnetic field strength H(t) and magnetic flux density B(t) can be obtained [11]. The output power of the excitation source, i.e., the magnetic core loss density P, can be calculated using Formula (1).

(1) $$P=\frac{1}{T}\int_{0}^{T}u(t)\cdot i(t)dt/(A_{e}\cdot l_{e})=\frac{1}{T}\int_{B(0)}^{B(T)}HdB$$

**Figure 2:**
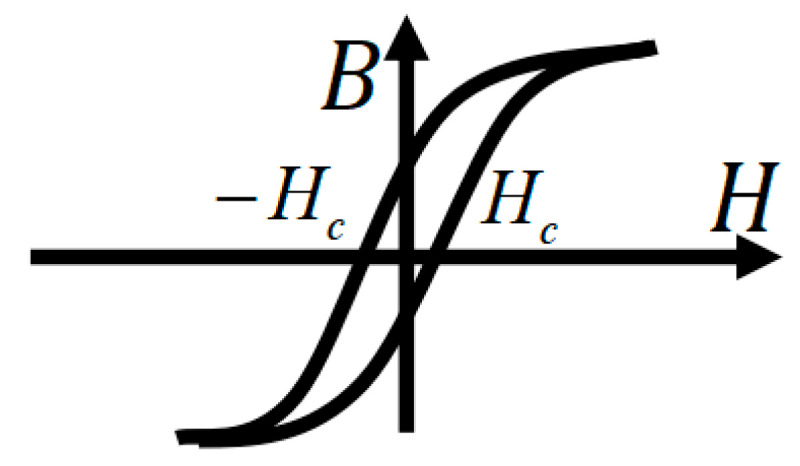
*B*-*H* hysteresis loop.

Formula (1) also indicates that the magnetic core loss per unit volume within one excitation cycle equals the area of the *B*-*H* hysteresis loop, as shown in Figure 2.

The magnetic core loss can be divided into three parts: hysteresis loss, eddy current loss, and residual loss [12]. This method of calculating magnetic core losses is called the loss separation model, which attempts to obtain the total loss by separately calculating the losses of these components. The calculation formula is as follows.

(2) $$P_{core}=P_{h}+P_{cl}+P_{e}$$

Here, $P_{core}$ is the total magnetic core loss density (also known as unit volume magnetic core loss, abbreviated as magnetic core loss, often abbreviated as P); $P_{h}$ is hysteresis loss; $P_{cl}$ is eddy current loss; and $P_{e}$ is the residual loss.

From the above, it can be seen that there are many factors that affect the loss of magnetic cores. At present, there are few universally applicable and high-precision models, which makes it difficult for the industry to accurately evaluate the loss of magnetic cores when using magnetic components, thereby affecting the evaluation of power converter efficiency. This study develops a core loss model for magnetic components using XGBoost with hyperparameters optimized by simulated annealing (SA). The model is trained and tested under operating conditions of 10 kHz–1 MHz frequency and 0.05–0.3 T flux density, with a target prediction error (MAPE) below 10%. The main contribution of this work is not a new learning algorithm, but a validated data-driven modeling framework specifically tailored for prediction of magnetic core loss, which has not been systematically studied in the previous literature.

## 2. Problem Description

**Figure 3:**
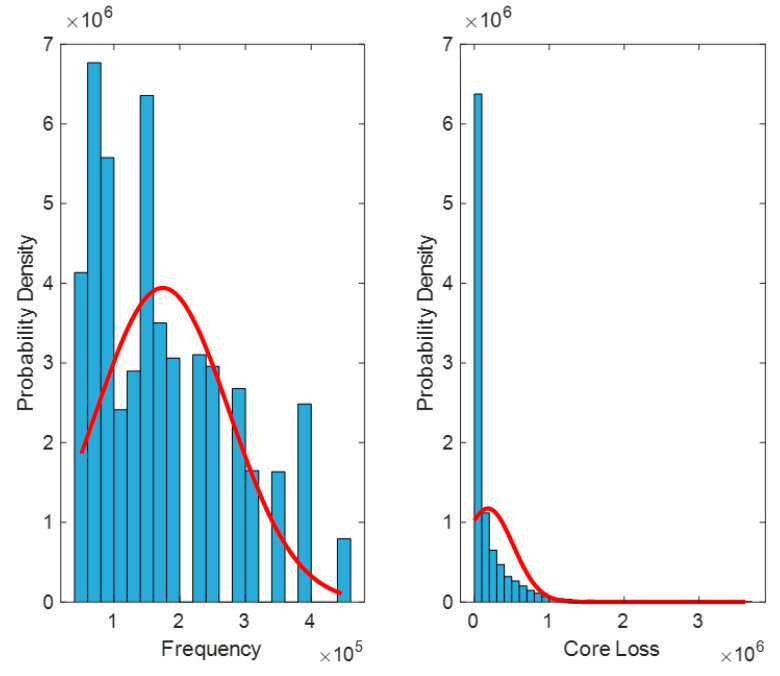
Normal distribution test of data.

A detailed introduction of the dataset is provided in the Supplementary Materials. To solve the problem of accurate calculation of magnetic core material loss in magnetic components, first use MATLAB R2024a’s find function to search for missing values in the dataset, and then perform a normal distribution test on the data to determine whether there are outliers. Outliers are typically defined as values that are less than Q1–1.5 IQR or greater than Q3 + 1.5 IQR (where Q1 is the first quartile, Q3 is the third quartile, and IQR is the interquartile range) [13]. In Figure 3, the unit of frequency is Hz, and the unit of magnetic core loss is W/m^3^.

Through visualization, it can be roughly seen that the data does not follow a normal distribution. In order to further verify, it is necessary to determine the distribution pattern of the data. Here, the Kolmogorov–Smirnov (K–S) test is used. The K–S test is a non-parametric statistical test method, most commonly used to test whether the dataset follows a normal distribution [14]. The basic principle is to compare the cumulative distribution function of the dataset with the theoretical distribution function, and determine whether the dataset conforms to the theoretical distribution by calculating the maximum difference between the two. If the maximum difference is less than a certain critical value, the dataset is considered to follow the theoretical distribution. The single sample K–S test is used to test whether the observed empirical distribution of a data is a known theoretical distribution [15].

**Figure 4:**
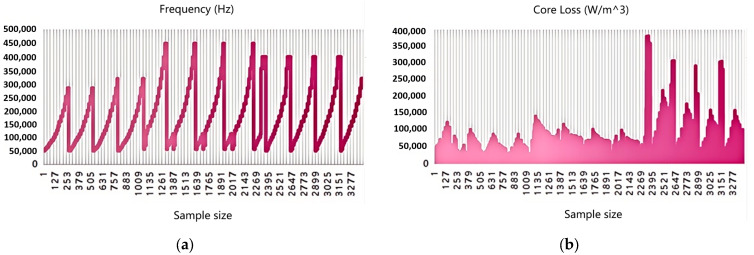
Distribution and trend of frequency and core loss: (**a**) Distribution and trend of frequency. (**b**) Distribution and trend of core loss.

In descriptive analysis, basic statistical measures such as mean, median, standard deviation, maximum, and minimum are usually calculated, and the distribution and trend of the data are presented through visualization methods. In order to present the specific situation of the data more intuitively, a visualization result was drawn using the processed data as shown in Figure 4. Figure 4 shows the trend of frequency and magnetic core loss as a function of sample size. The horizontal axis of Figure 4 represents the sample size, and the interval of sample size is 126.

Figure 4a shows the variation of frequency with sample number. The frequency exhibits periodic fluctuations, with some frequencies reaching high values close to 500,000 Hz, indicating that, under different experimental conditions, the frequency covers a very wide range and the variation from low to high frequency is well performed. Figure 4b shows the trend of magnetic core loss values for each sample. It can be clearly seen that when the sample number is low, the loss is small and relatively stable, while when the sample number increases, the magnetic core loss rapidly increases and reaches the order of millions. The increase in sample loss in the later stage may be related to specific materials, waveforms, or operating conditions.

For a magnetic flux density data matrix X, its size is m×n. m represents the number of samples, that is, the number of experimental data collected from different materials under different excitation waveforms, and n represents the length of the magnetic flux density sequence for each sample.

(3) $$X=\left[\begin{array}{cccc} x_{11} & x_{12} & \ldots & x_{1n}\\ x_{21} & x_{22} & \ldots & x_{2n}\\ \vdots & \vdots & \ddots & \vdots \\ x_{m1} & x_{m2} & \ldots & x_{mn} \end{array}\right]$$

$X_{ij}$ represents the magnetic flux density value of the i-th sample at the j-th time point. The excitation waveform in the experimental data is a multi-class variable. In order to train the classification model, this paper converts it into numerical form: $y_{i}$ represents the waveform category of the i-th sample. The categories are defined as: y=1 (sine wave), y=2 (triangular wave), and y=3 (stair wave). eXtreme Gradient Boosting (XGBoost) is an efficient and popular open-source machine learning library designed for accurate supervised learning (regression, classification, ranking etc.). In order to meet the requirements of the XGBoost model, further adjust the labels to count from 0.

This article effectively classifies different waveforms by extracting key features from both time and frequency domains. Time domain features are directly extracted from the magnetic flux density time series of each sample. Assuming the time series of the i-th sample is $x_{i}=\{x_{i1},x_{i2},\ldots ,x_{in}\}$, first, we need to extract the time domain features of the excitation waveform; time domain features include peak, mean, standard deviation, and so on, of the waveform. Then, after obtaining these features, the above feature selection method can be used for screening, and a classification model can be established to evaluate the effectiveness of the classification model.

The mean value $u_{i}$ reflects the average level of magnetic flux intensity of the sample, and $u_{i}$ represents the central trend of the magnetic flux density of the i-th sample [16]. The mean is the average value of the excitation waveform within one cycle, which can be used to evaluate the DC component of the waveform and help determine whether there is a DC bias in the magnetic core. The calculation formula for $u_{i}$ is as follows.

(4) $$u_{i}=\frac{1}{n}\sum_{j=1}^{n}x_{ij}$$

The standard deviation $\sigma_{i}$ is a core indicator in statistics that measures the degree of dispersion of data distribution. Its mathematical definition is the square root of the average of the squared differences between each data point and the mean. The larger the standard deviation, the greater the deviation of data points from the mean and the more dispersed the data distribution. The smaller the standard deviation, the more concentrated the data is around the mean distribution. The calculation formula for $\sigma_{i}$ is as follows.

(5) $$\sigma_{i}=\sqrt{\frac{1}{n}{\sum_{j=1}^{n}\left(x_{ij}-u_{i}\right)}^{2}}$$

Peak value is the maximum value reached by the excitation waveform within one cycle. It reflects the maximum amplitude of the waveform and is of great significance for evaluating the saturation level and potential losses of the magnetic core. The maximum and minimum values are $\max(x_{i})$ and $\min(x_{i})$, respectively.

(6) $$\max(x_{i})=\underset{j\in [1,n]}{\max}(x_{ij}),\min(x_{i})=\underset{j\in [1,n]}{\min}(x_{ij})$$

These features demonstrate the amplitude differences of sine waves, triangular waves, and trapezoidal waves. The $skewness_{i}$ measures the asymmetry of a waveform [17].

(7) $$skewness_{i}=\frac{1}{n}\overset{n}{\underset{j=1}{\sum }}{\left(\frac{x_{ij}-u_{i}}{\sigma_{i}}\right)}^{3}$$

The $smoothness_{i}$ measures the stability of the waveform. The higher the smoothness, the smoother the waveform changes, and the more it can reflect the uniformity of magnetic flux density [18].

(8) $$smoothness_{i}=\overset{n}{\underset{j=2}{\sum }}|x_{ij}-x_{i(j-1)}|$$

The $slope_{i}$ reveals the trend of waveform changes, with positive values indicating a gradual increase in magnetic flux density and negative values indicating a gradual decrease in magnetic flux density.

(9) $$slope_{i}=\frac{x_{in}-x_{i1}}{n}$$

Time domain features can reflect the overall shape and trend of waveform changes. Beyond the time domain, the frequency domain is another key to revealing the waveform structure. Fourier transform can see the oscillation frequency of magnetic flux density and extract key information. By using the Fourier transform formula $F(x_{i})$, we can convert the time signal into a frequency domain signal. The expression for $F(x_{i})$ is shown below.

(10) $$F(x_{i})=\overset{n}{\underset{j=1}{\sum }}x_{ij}e^{-i2\pi jk/n}$$

The main frequency $L_{i}$ is the strongest oscillation frequency of the energy star in the waveform, which directly tells us the main vibration mode of this waveform. The expression for $L_{i}$ is as follows.

(11) $$L_{i}=\arg\max(|F(x_{i})|)$$

The high-frequency energy ratio $S_{i}$ reveals the energy proportion of high-frequency components in the waveform [19]. The more concentrated the energy, the more regular the waveform. The expression for $S_{i}$ is as follows.

(12) $$S_{i}=\frac{\overset{10}{\underset{k=2}{\sum }}|F{\left(x_{i}\right)}_{k}|}{\overset{n}{\underset{k=1}{\sum }}|F{\left(x_{i}\right)}_{k}|}$$

Spectral entropy $G_{i}$ is the ultimate indicator of waveform complexity. It tells us that the more complex the distribution of spectral energy, the more complex the waveform changes. The expression for $G_{i}$ is as follows.

(13) $$G_{i}=-\overset{n}{\underset{k=1}{\sum }}P_{k}\log P_{k},P_{k}=\frac{\left|F{\left(x_{i}\right)}_{k}\right|}{\overset{n}{\underset{k=1}{\sum }}|F{\left(x_{i}\right)}_{k}|}$$

In order to help simplify the construction of the model and ensure the feasibility of the analysis, the following basic assumptions are made:

(1) Single factor independent assumption: When studying the effect of a single factor on magnetic core loss, other factors (such as temperature, material, waveform, etc.) remain constant.

(2) Assumption of linear working range of magnetic core material: The working range of magnetic core material is linear, not saturated, which limits the range of action of magnetic core material.

(3) Periodic assumption of magnetic flux density: Magnetic flux density has periodicity, that is, the internal magnetic flux density of the magnetic core undergoes periodic changes over time and maintains a stable fluctuation within a certain period.

## 3. Model Construction

XGBoost is an ensemble learning method based on decision trees, using gradient boosting as a framework, aiming to efficiently implement the Gradient Boosting Decision Tree (GBDT) algorithm and perform multiple optimizations [20]. Its core idea is similar to GBDT, but through the introduction of second-order derivative information, regularization term control of model complexity, and parallel computing strategy, the accuracy and speed have been improved. XGBoost is widely used in supervised learning tasks such as classification and regression, particularly in the fields of data mining and recommendation systems [21].

The basic idea of XGBoost is to iteratively construct a decision tree by minimizing the loss function L(θ) [22]. The form of the loss function L(θ) is:

(14) $$L(\theta )=\overset{m}{\underset{i=1}{\sum }}l(y_{i},\hat{y_{i}})+\overset{T}{\underset{t=1}{\sum }}\Omega (f_{t})$$

Here, $l(y_{i},\hat{y_{i}})$ is the prediction errors of sample i, $\Omega (f_{t})$ is the penalty term for model complexity, T is the number of decision trees, and θ is the model parameter. In each iteration, XGBoost updates the model by fitting residuals.

(15) $${\hat{y_{i}}}^{\left(t+1\right)}={\hat{y_{i}}}^{\left(t\right)}+\eta f_{t}(x_{i})$$

η is the learning rate, and $f_{t}(x_{i})$ is the predicted result of the t-th tree. The regularization term $\Omega (f_{t})$ is used to control the complexity of the XGBoost model, thereby preventing overfitting.

(16) $$\Omega (f_{t})=\gamma T+\frac{1}{2}\lambda \sum_{j=1}^{T}w_{j}^{2}$$

γ and λ are regularization parameters used to control the complexity of the tree. The advantage of XGBoost is that it can not only handle high-dimensional data, but also accelerate model training through efficient algorithm optimization. For the classification problem of excitation waveforms, XGBoost’s powerful ability to capture complex nonlinear relationships makes it an excellent classification tool. Classify waveforms using XGBoost and establish a confusion matrix between actual and predicted waveforms to evaluate the classification and prediction performance of XGBoost. The hyperparameters were set as follows: learning rate = 0.001, number of trees = 500, maximum depth = 6, and regularization term = 0.1. These values were determined via 5-fold cross-validation on the training set.

By using the confusion matrix, we can calculate various performance metrics such as Precision, Recall, and F1−Score. Precision focuses on how many samples predicted as positive are truly positive, while Recall focuses on how many of all truly positive samples are predicted as positive by the model. The F1−Score is the harmonic mean of Precision and Recall, used to comprehensively evaluate the performance of a model [23]. Specifically, the F1−Score is obtained by calculating the harmonic average of the Precision and Recall, where the harmonic average is the product of two numbers divided by their sum to balance the impact of the Precision and Recall on the model performance, so as to obtain a comprehensive evaluation index. The formula for F1−Score is:

(17) $$F1-Score=\frac{2\times TP}{2\times TP+FP+FN}$$

The formula for calculating the Precision of the confusion matrix is:

(18) $$Precision=\frac{TP}{TP+FP}$$

Precision is used to evaluate the accuracy of the model in predicting positive cases [24]. Among them, TP (number of true cases) represents the number of samples correctly predicted by the model as positive cases, and TP (number of false positive cases) represents the number of samples incorrectly predicted by the model as positive cases. The range of Precision values is from 0 to 1. The closer the value is to 1, the higher the accuracy of the model in predicting positive cases. The closer the value is to 0, the lower the accuracy. Recall in the confusion matrix is:

(19) $$Recall=\frac{TP}{TP+FN}$$

**Figure 5:**
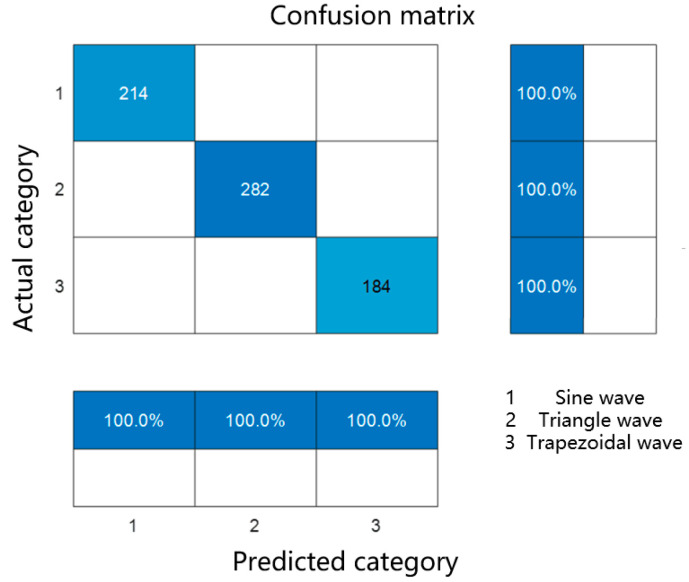
Confusion matrix of actual type and predicted type.

Recall is used to measure the classifier’s ability to correctly identify positive samples [25]. Specifically, Recall refers to the proportion of samples that are correctly predicted as positive in reality. In the confusion matrix, TP represents the number of samples that are actually positive and predicted to be positive, while FN represents the number of samples that are actually positive but predicted to be negative. Therefore, the closer the Recall of the confusion matrix is to 1, the stronger the model’s ability to identify positive samples. When the model is trained for classification, the real classification results in the sample will be compared with the predicted classification results trained by the model. We divided the data into training and testing sets to validate the model. Figure 5 shows the confusion matrix between the XGBoost model’s classification prediction results and the actual classification results.

According to the confusion matrix in Figure 5, it can be concluded that the waveform classification of the XGBoost model is consistent with the actual waveform classification results. After running the confusion matrix code, it was found that its Precision, Recall, and F1−Score were all 1, reflecting the excellent classification prediction performance of the established model.

The quantities of the three types of waveforms in the dataset are shown in Table 1.

The classification results of sample numbers 5, 15, 25, 35, 45, 55, 65 and 75 in the dataset are shown in Table 2.

In magnetic components, core loss is influenced by multiple factors, among which temperature, excitation waveform, and material are important independent factors. A thorough analysis of these factors can help to more accurately model and optimize the performance of magnetic cores. The experimental dataset includes material types (Material 1, Material 2, Material 3, Material 4), temperatures (25 °C, 50 °C, 70 °C, 90 °C), excitation waveforms (sine wave, triangle wave, and trapezoidal wave), and magnetic core losses.

One-way ANOVA is used to compare the mean differences of three or more independent groups to test whether a dependent variable (usually a continuous variable) has significant mean differences between different categories or groups (defined by a single independent variable) [26]. This method is commonly used to detect whether there is a significant difference in the mean of a dependent variable between different categories or groups. Its basic theory is based on the idea of analysis of variation, which decomposes the total variation (i.e., the variation of all observed values) into two parts: inter group variation and intra group variation. Inter group variation reflects the difference in mean values between different groups, while intra group variation reflects individual differences within the same group. The key to ANOVA lies in comparing the relative size of inter group variation and intra group variation: if inter group variation is significantly greater than intra group variation, it indicates a significant difference in the mean values between groups [27].

**Figure 6:**
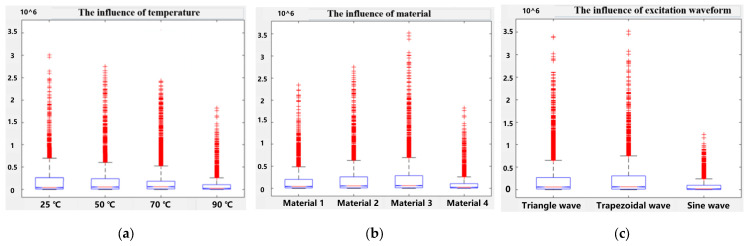
Magnetic core loss box wiring diagram: (**a**) The influence of temperature. (**b**) The influence of material. (**c**) The influence of excitation waveform.

This method is also known as the F-test, or F-ratio. It is mainly used to analyze the situation when only one independent variable changes, and to test whether the difference between the means of each group is statistically significant by calculating the value of the F-statistic. If the *p*-value is greater than the critical value corresponding to a certain significance level, the null hypothesis can be rejected and it can be assumed that there is a significant difference between the means of each group [28]. The *p*-value is obtained based on the significance test method and is used to evaluate whether the observed effect may be caused by chance (i.e., sampling error). The magnitude of the *p*-value reflects the degree of difference between the observed effect and the expected random fluctuations. Based on the magnitude of the *p*-value, it can be determined whether the observed effect has statistical significance, that is, whether it is significant enough to not be simply attributed to random error. Generally, *p* < 0.05 is considered statistically significant, *p* < 0.01 is considered statistically significant, and *p* < 0.001 is considered extremely significant [29]. After significance testing, p_t_ = 0.018, p_m_ = 0.025, and p_w_ = 0.021. Due to the fact that p_t_, p_m_, and p_w_ are all less than 0.05, it is believed that single factors such as temperature, material, and waveform have an impact on magnetic core loss. The box plot of magnetic core loss is shown in Figure 6.

When the temperature is 90 °C, the magnetic core loss is the smallest, followed by 70 °C, then 50 °C, and the largest loss is 25 °C. As the temperature increases, the magnetic core loss tends to decrease. Material 4 has the smallest magnetic core loss, followed by material 1, material 2, and material 3 which has the largest loss. The magnetic core loss is minimized when the excitation waveform is a sine wave, followed by a triangle wave, and then a trapezoidal wave.

Choose a linear regression model for fitting, with the following structure: “core loss = §0 + §1 · material + §2 · temperature + §3 · waveform + §4 · (material · temperature) + §5 · (temperature · waveform) + §6 · (material · waveform) + ε”. The core variables in the model include independent effects of material, temperature, and waveform, as well as interaction terms between them, such as interaction between temperature and waveform, interaction between material and waveform, and interaction between material and temperature. Measure the degree of influence of each variable on magnetic core loss through the coefficient § in the linear regression model. In regression analysis, the response variable is magnetic core loss in W/m^3^, while the independent variables are temperature, material, and waveform. The regression model results are obtained by linear regression fitting the dataset, and the coefficients and significance levels of the model can help understand the independent effects and interactions of different factors. The regression coefficient § in the model represents the magnitude of the impact of the corresponding factors on the magnetic core loss.

“Synergistic” means “working together”, and synergistic effect refers to the overall effect produced when two or more factors work together. In the process of analyzing magnetic core losses, there are often multiple factors that interact with each other. Analyzing the synergistic effects of temperature, material, and excitation waveform can provide a more comprehensive understanding of the complex mechanism of magnetic core loss.

The formula for the interaction between temperature and materials is:

(20) $$P_{T,M}=\frac{1}{n_{t,m}}\Sigma_{k=1}^{n}P_{k}$$

The formula for the interaction between material and excitation waveform is:

(21) $$P_{M,W}=\frac{1}{n_{m,w}}\Sigma_{k=1}^{n}P_{k}$$

The formula for the interaction between temperature and excitation waveform is:

(22) $$P_{T,W}=\frac{1}{n_{t,w}}\Sigma_{k=1}^{n}P_{k}$$

Here, $n_{t,w}$ represents the number of samples under specific temperature T and waveform W conditions, while $P_{T,W}$ is the core loss. Due to p_t,w_ = 0.019 < 0.05, it was found that there is a significant synergistic effect between temperature and excitation waveform; p_t,m_ = 0.732 > 0.05. The synergistic effect between temperature and material was found to be not significant enough; p_m,w_ = 0.001 << 0.05. The synergistic effect between the material and excitation waveform was found to be very significant.

Table 3 shows the effects of three excitation waveforms (triangle wave, sine wave, trapezoidal wave) on magnetic core losses at different temperatures. The values in Table 3 represents the average magnetic core loss in units of kW/m^3^. Error bars represent ±1 standard deviation calculated from three repeated measurements under identical conditions.

**Figure 7:**
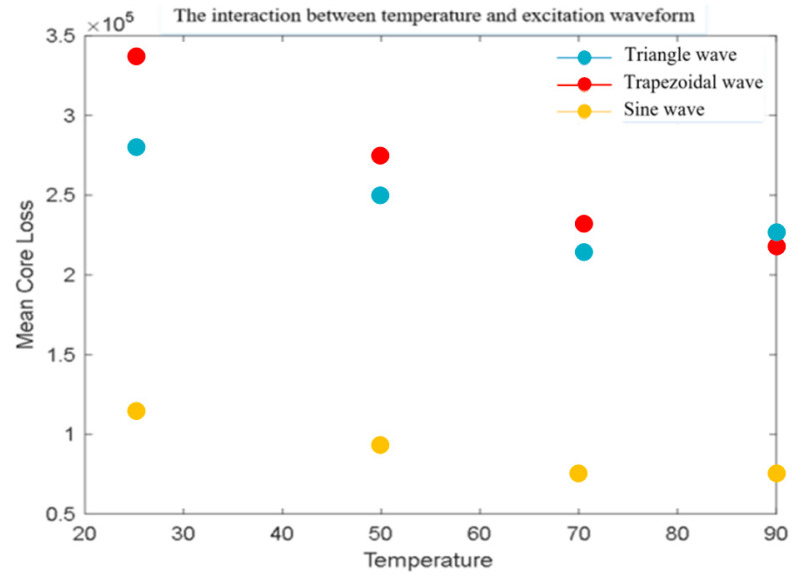
Interaction between temperature and excitation waveform.

The visual representation of the mean magnetic core loss in Table 3 is shown in Figure 7.

The main observation of Figure 7 is that the core losses of the three waveforms gradually decrease with increasing temperature. Overall, the higher the temperature, the lower the loss. Overall, sine waves perform the best, followed by trapezoidal waves and triangle waves. As the temperature increases, although the difference between triangle waves and trapezoidal waves gradually narrows, sine waves always dominate.

Table 4 shows the magnetic core losses of various materials at different temperatures. The values in Table 4 represents the average magnetic core loss in units of kW/m^3^.

**Figure 8:**
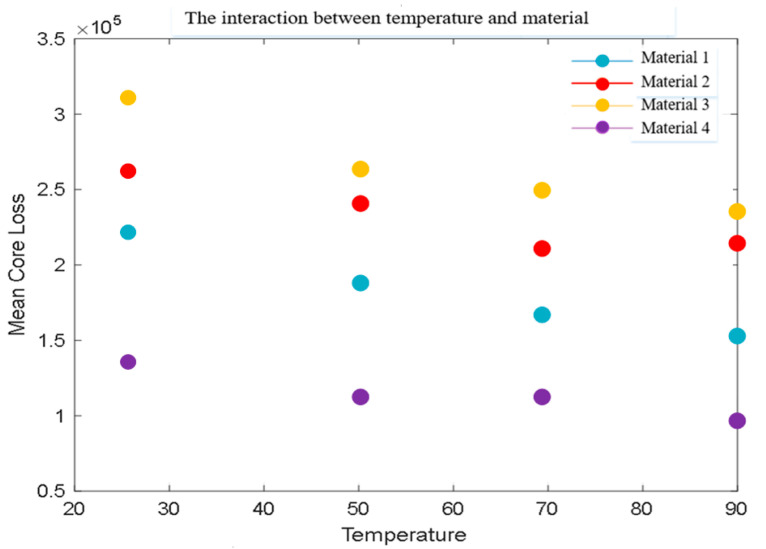
Interaction between temperature and material.

The visual representation of the mean magnetic core loss in Table 4 is shown in Figure 8.

The main observations in Figure 8 are as follows: the magnetic core losses of the four materials gradually decrease with increasing temperature, and overall, the higher the temperature, the lower the losses. Material 4 performs the best at all temperatures, especially in high-temperature environments where losses are significantly lower than other materials. Material 1 also exhibits good performance under high temperature conditions, while materials 2 and 3 have relatively high losses.

Table 5 shows the effects of three excitation waveforms on magnetic core losses under different materials. The values in Table 5 represent the average magnetic core loss in units of kW/m^3^.

**Figure 9:**
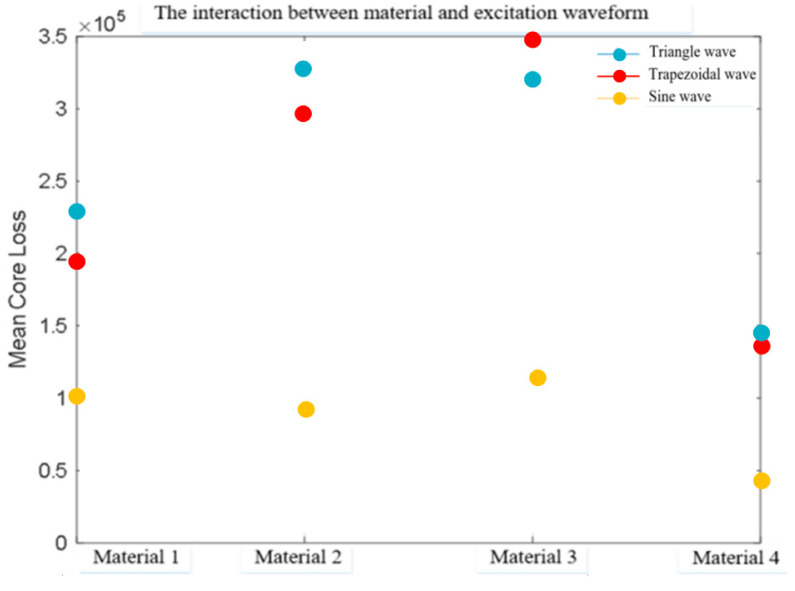
Interaction between material and excitation waveform.

The visual representation of the mean magnetic core loss in Table 5 is shown in Figure 9.

The following is the main observation and analysis of Figure 9: material 4 performs better than other materials in all waveforms, especially in triangular and sine waves, with significantly lower losses than other materials. Material 1 performs well, especially under sine waves. Material 3 performs the worst, with severe losses under trapezoidal waveforms.

In an ideal situation, by precisely controlling temperature and waveform, and selecting high-purity and uniform magnetic core materials, it is possible to comprehensively understand the independent and comprehensive effects of different factors on magnetic core losses. This research under pure theoretical conditions helps to promote the optimization of magnetic core losses in practical applications, providing a theoretical basis and data support for designing more efficient magnetic components. Through the prediction of the regression model, we found the minimum value of the total loss of the magnetic core under different combination conditions. According to the prediction results of the model, the conditions for achieving the minimum loss under the optimal conditions are as follows: 90 °C, Sine wave and Material 4.

Establish a prediction model for magnetic core loss through XGBoost regression, then predict existing data and compare the predicted results with actual values to evaluate the prediction model. Firstly, the dataset is divided into two parts: the training set and the testing set. We take magnetic core material, temperature, excitation waveform, frequency, and peak magnetic flux density as independent variables X, and magnetic core loss as dependent variable Y. At the same time, we specify that the test set should include 30% of the samples in the original datasets X and Y. Therefore, the remaining 70% of the samples will be used as the training set. Train the model using the training set, and use the segmented test set to predict the magnetic core loss. Finally, various evaluation metrics were calculated based on Y’s training and testing sets.

The proposed model demonstrates consistent accuracy under the following tested conditions: frequency range 10 kHz–1 MHz, flux density 0.05–0.3 T, temperature 25–100 °C. The evaluation system mainly includes the following indicators.

(1) Determination coefficient R^2^

The closer R^2^ is to 1, the better the fit of the model to the data, that is, the smaller the difference between the predicted and actual values of the model, and the more variability the model can explain [30]. When R^2^ is equal to 1, it indicates that the model fits the data perfectly. When R^2^ is equal to 0, it indicates that the model has failed to explain any variation. In regression analysis, R^2^ is used to evaluate the degree of agreement between the model’s predicted values and actual values. The higher the R^2^, the better the fitting effect of the model, which can better explain the variation of the dependent variable. R^2^ is an important indicator for evaluating the goodness of fit of the model.

(2) Mean absolute percentage error (MAPE)

MAPE is an indicator for measuring prediction accuracy, which evaluates the performance of a model by calculating the percentage error between predicted and actual values [31]. Its main advantage is that it is simple and easy to understand, and can intuitively reflect the size of prediction errors. The formula is:

(23) MAPE=(∑((X−Y)/X)×100%)/N

In the formula, X represents the measured value, Y represents the simulated value, and N represents the total number of samples.

(3) Mean squared error (MSE)

MSE is an intuitive error measurement method that reflects the overall discrepancy between predicted and actual values [32]. In many cases, MSE can serve as an optimization objective, improving model prediction accuracy by minimizing MSE. MSE is widely used in statistics and machine learning to evaluate model prediction accuracy. For example, in regression analysis, MSE measures the difference between model predictions and actual observations; in neural network training, MSE acts as a loss function to optimize model parameters and reduce prediction errors. The formula is:

(24) $$MSE\;=\;1/n\times \Sigma {\left(y_{i}-{\hat{y}}_{i}\right)}^{2}$$

(4) Mean absolute error (MAE)

MAE is an indicator used to measure the difference between predicted values and actual values [33]. It quantifies the magnitude of error by calculating the average of the absolute differences between predicted and actual values. The formula is:

(25) $$MAE\;=\;1/n\times \Sigma \left|y_{i}-{\hat{y}}_{i}\right|$$

The predictive performance indicators of the XGBoost regression model are: R^2^ = 0.9703, MAPE = 3.6251%, MSE = 89.0471, MAE = 20.5034. All indicators perform well, indicating that the model has good predictive performance.

## 4. Model Solving

The focus of this study is to achieve effective knowledge transfer. Specifically, the aim is to transfer diagnostic knowledge with rich labels and clear physical meanings from the source domain (bearing test bench) to the target domain (train operation data) with fuzzy fault types, significant noise, and scarce samples. Therefore, the technical key lies in designing a migration mechanism that can overcome the inter domain differences mentioned above, ultimately enabling the model to have the ability to identify unknown faults in the target domain.

**Figure 10:**
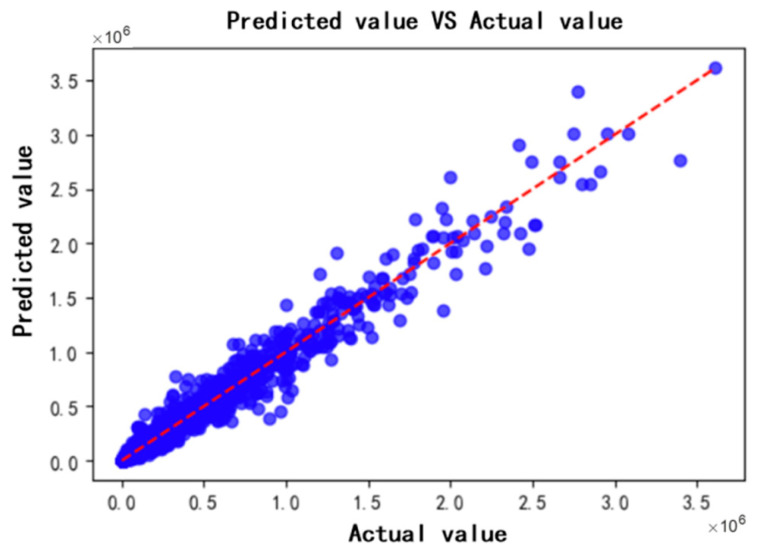
Fit between actual and predicted values.

Next, the XGBoost regression model is used to predict the data and form a fitting effect graph. The fitting between the true value and the predicted value is shown in Figure 10.

The red dashed line in Figure 10 represents the distribution of predicted values, which is the diagonal of the ideal situation. The predicted value is calculated through a model and used to predict a certain outcome or output. The blue dots represent the true values, which are the actual observed data points. From the above figure, we can clearly see that the predicted values closely follow the distribution of the true values, so it can be considered that the model’s predictions are relatively accurate.

**Figure 11:**
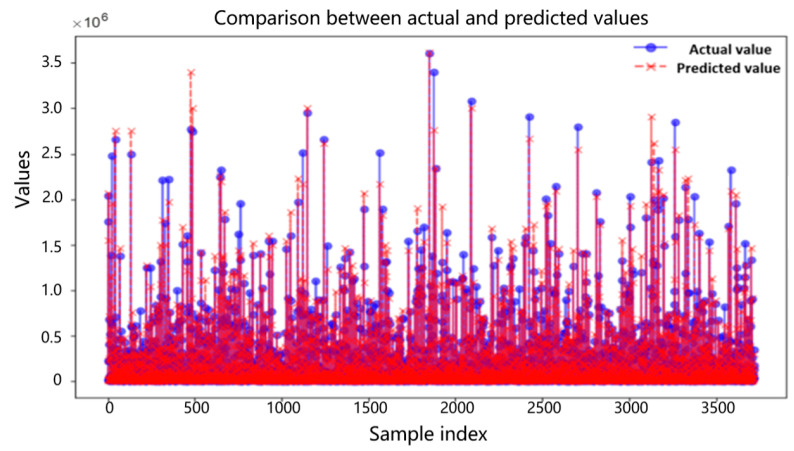
Numerical comparison between actual and predicted values.

The residual is the difference between the predicted value and the true value, and an ideal residual graph should follow a normal distribution [34]. The numerical comparison between the actual value and the predicted value is shown in Figure 11.

Figure 11 shows the numerical comparison between the predicted values and the true values. It is evident from the figure that although there are some significant differences between the predicted values and the true values, the predicted values mostly vary within a certain range near the true values. Therefore, in summary, it can be seen that this prediction model is basically accurate.

In the design and optimization of magnetic devices, core loss and transmission of magnetic energy are two important performance indicators [35]. Expect to minimize magnetic core losses and increase transmission magnetic energy as much as possible under different working conditions. The setting of constraints is crucial for the study of minimizing magnetic core losses and maximizing magnetic energy transmission. Different constraint conditions directly affect the optimization results of the model, thereby determining the actual performance of the magnetic core. Core loss is the energy loss caused by magnetization or demagnetization during the operation of magnetic components. Reducing magnetic losses is a key issue in improving device performance. The transmission of magnetic field energy is a key parameter for measuring the efficiency of electromagnetic energy conversion, with the aim of maximizing the transmitted magnetic energy.

The objective function of this article is measured by calculating the transmitted energy and core loss. The purpose is to maximize the transmission of magnetic energy while minimizing core loss.

(26) $$Y(temperature_{idx},material_{idx},wave_{idx},f,B)=\frac{Core\;Loss}{Transmitted\;Energy+\varepsilon }$$

**Figure 12:**
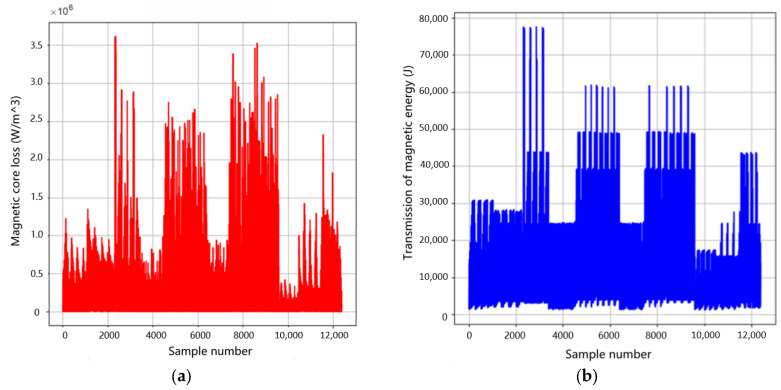
Diagram of magnetic core loss and transmission of magnetic energy: (**a**) Changes in magnetic core loss. (**b**) Changes in transmission of magnetic energy.

B is the peak value of magnetic flux density, f is frequency, and ε is a very small number to prevent the denominator from being zero. There are many methods to solve multi-objective optimization problems, such as the ideal point method, the multi-objective weighted calculation method, and the main objective and constraint method. For example, evolutionary computation methods such as genetic algorithm, particle swarm optimization, ant colony algorithm, etc. can search and generate solutions on the Pareto front in the solution space. Specifically, multi-objective evolutionary algorithms are specially designed to handle multi-objective optimization problems and can directly explore and maintain Pareto frontiers in the solution space [36]. The experimental data was processed using multi-objective optimization theory, and Figure 12 shows the changes in magnetic core loss and transmitted magnetic energy.

In practical applications, the selection of appropriate optimization strategies and algorithms usually depends on the specific characteristics of the problem, the properties of the objective function, and the decision-maker’s preference for balancing different objectives. The purpose of multi-objective optimization is to provide a set of solutions, from which decision-makers can choose the most suitable one based on their actual situation and preferences. Therefore, multi-objective optimization is not only a technical problem, but also a process involving decision-makers’ judgment and selection.

Simulated annealing algorithm is widely used in various optimization problems due to its powerful global search ability and adaptability [37]. It exhibits good performance in solving complex multi-objective optimization problems, especially when dealing with nonlinear and non-convex problems, and can effectively avoid the trap of local optimal solutions. Based on the specific situation of magnetic core loss and transmission of magnetic energy, it was decided to use simulated annealing algorithm to solve this problem. The simulated annealing algorithm mainly includes the following steps.

① Initialization: Temperature (temp_idx), material (mat_idx), waveform (wave_idx), frequency (freq_idx), peak magnetic flux density (flux_density). Among them, a value is randomly generated within the specified frequency range [freq-min, freq-max], and the peak magnetic flux density (flux_density) is also randomly generated within the specified peak magnetic flux density range [flux_density-min, flux_density-max]. The temperature, material, and waveform are randomly generated list index values.

② Generate a new solution: Randomly selecting a dimension (such as frequency, material, waveform, temperature, or peak magnetic flux density) in the model and making appropriate changes to it will generate a new solution. The new solution formula is as follows.

(27) $$X_{new}=X_{current}+\zeta$$

Here, ζ is the change value of a certain factor.

③ Solving the objective function: Calculate the objective function values $f(X_{current})$ and $f(X_{new})$ for the current solution $X_{current}$ and the new solution $X_{new}$.

④ Compare the function values of newer solutions: If $f(X_{new})$ < $f(X_{current})$, accept the new solution. If $f(X_{new})$ > $f(X_{current})$, the new solution is accepted with a certain probability, which is calculated by the Boltzmann distribution law. The formula is:

(28) $$P=\exp(-\frac{\Delta E}{T})$$

T represents the current temperature, and the energy difference is ΔE = $f(X_{new})$−$f(X_{current})$. When ΔE > 0, the simulated annealing algorithm will use probability P to determine whether to accept the new solution. If the random number *n* satisfies n < P, the new solution is accepted.

⑤ Cool down: Lowering the temperature according to certain cooling rules is the core of the simulated annealing algorithm. The temperature drop reduces the acceptance probability of the simulated annealing algorithm for inferior solutions, until the algorithm can only accept better solutions in the end. The updated temperature formula is as follows.

(29) $$T_{new}=C\times T_{current}$$

After each iteration, the temperature will be updated according to this formula. C is the temperature attenuation coefficient, which generally ranges from 0 to 1. $T_{current}$ represents the current temperature.

⑥ Determine termination conditions: If the termination condition is met (such as not accepting a new solution for several consecutive times), the algorithm ends. The termination condition is that the current temperature drops to the set minimum temperature or the maximum number of iterations is completed at each temperature.

**Figure 13:**
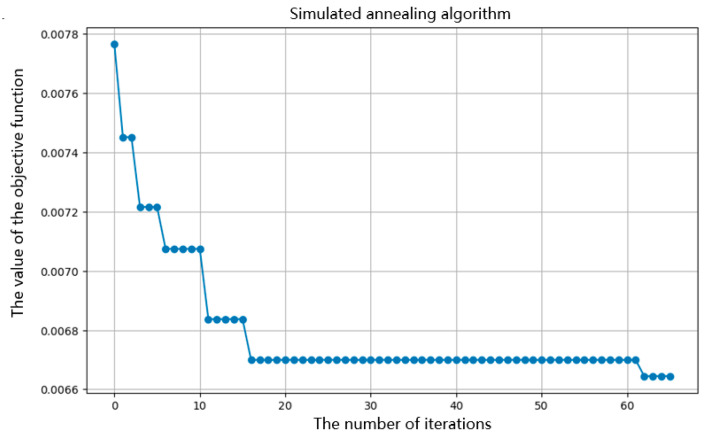
Simulated annealing algorithm optimization process diagram.

Figure 13 shows the optimization process of the simulated annealing algorithm.

The establishment of the simulated annealing algorithm model requires the introduction of constraints. In the modeling process, multi-objective optimization methods can be used to solve the objective function through simulated annealing algorithm. Set initial parameters and define the temperature change pattern, gradually approaching the optimal solution through multiple iterations. The process of simulated annealing can effectively avoid getting stuck in local optima, thereby improving global search capabilities.

Based on the set objective function and constraints, obtain an effective model that can maximize the transmission of magnetic energy under given conditions. This process not only emphasizes the importance of theoretical analysis, but also verifies the effectiveness of model construction in practical applications. Through the feedback of experimental and simulation results, the model parameters can be further optimized to better meet the actual engineering requirements.

From the above figure, it can be seen that the simulated annealing algorithm has found the optimal solution, as the objective function value is continuously decreasing. According to the Formula (26), this indicates that the ratio of core loss to transmitted energy is continuously decreasing, which means that the multi-objective of minimizing core loss and maximizing transmitted magnetic energy can be achieved. The results are shown in Table 6.

From Table 6, it can be seen that when the temperature is 70 °C the material is material 1 and the excitation waveform is a sine wave; the objective function obtains the optimal solution, and the ratio of magnetic core loss to transmitted magnetic energy reaches the minimum.

Future research directions that can be considered include: (1) exploring the impact of more complex interactions on magnetic core losses, such as the three-factor interaction between temperature, material, and excitation waveform. (2) This study can also be extended to the loss control of other magnetic devices with similar magnetic core structures, studying the power consumption optimization problems of multiple systems such as motors and inductors. (3) Introduce more efficient intelligent solving algorithms.

## 5. Conclusions

The magnetic core loss referred to in this study denotes the power loss generated by magnetic materials under high-frequency alternating magnetic flux. The AC power method is employed to measure magnetic core loss. By collecting experimental data on core material losses of magnetic components under specified operating conditions (varying temperatures, frequencies, and magnetic flux densities), mathematical modeling (or algorithmic methods) are utilized to establish a loss model for power magnetic components. This model is then applied to predict core losses under other operational conditions, with the accuracy of the model being validated through testing.

The model established in this article considers many important factors, such as temperature, excitation waveform, magnetic core material, magnetic core loss, and transmission of magnetic energy. The model obtained in this way is in line with reality and has high application value. The model utilizes the ideas of comparative experiments and fitting evaluation to identify the important factors affecting magnetic core loss, transforming complex multi-objective problems into simple single objective problems. The main conclusions drawn from this study are as follows: (1) When the control material and excitation waveform remain unchanged, the magnetic core loss is minimized at a temperature of 90 °C, followed by 70 °C, then 50 °C, and the highest loss is at 25 °C. As the temperature increases, the magnetic core loss tends to decrease. (2) Keeping the temperature and material constant, the magnetic core loss is minimized when the excitation waveform is a sine wave, followed by a triangular wave, and then a trapezoidal wave. (3) Due to p_t,w_ = 0.019 < 0.05, it was found that there is a significant synergistic effect between temperature and excitation waveform; p_t,m_ = 0.732 > 0.05. The synergistic effect between temperature and material was found to be not significant enough; p_m,w_ = 0.001 << 0.05. The synergistic effect between the material and excitation waveform was found to be very significant.

## Figures and Tables

**Table 1:** Quantity of various types of three waveforms.

Category	Sine Wave	Triangle Wave	Trapezoidal Wave
Predicted quantity	214	282	184
Actual quantity	214	282	184

**Table 2:** Classification results of sample numbers.

Sample Serial Number	5	15	25	35	45	55	65	75
Classification result	Triangle wave	Sine wave	Triangle wave	Trapezoidal wave	Trapezoidal wave	Triangle wave	Triangle wave	Triangle wave

**Table 3:** Core loss values under different temperatures and excitation waveforms.

	Triangle Wave	Trapezoidal Wave	Sine Wave
**25 °C**	282 ± 2.36	342 ± 4.23	113 ± 2.04
**50 °C**	251 ± 2.01	278 ± 2.69	95 ± 0.85
**70 °C**	215 ± 3.45	232 ± 2.11	50 ± 0.29
**90 °C**	227 ± 2.24	221 ± 1.86	47 ± 1.37

**Table 4:** Core loss values under different temperatures and materials.

	Material 1	Material 2	Material 3	Material 4
**25 °C**	220 ± 2.35	261 ± 2.99	315 ± 3.56	142 ± 3.26
**50 °C**	186 ± 2.43	245 ± 2.65	266 ± 4.31	114 ± 4.11
**70 °C**	167 ± 1.47	210 ± 3.34	252 ± 2.44	115 ± 4.59
**90 °C**	153 ± 1.12	215 ± 3.79	237 ± 4.63	96 ± 3.98

**Table 5:** Core loss values under different materials and excitation waveforms.

	Triangle Wave	Trapezoidal Wave	Sine Wave
**Material 1**	232 ± 4.69	193 ± 1.82	101 ± 3.09
**Material 2**	331 ± 5.88	296 ± 2.58	95 ± 2.84
**Material 3**	325 ± 5.92	348 ± 5.61	119 ± 4.97
**Material 4**	149 ± 2.43	141 ± 2.36	47 ± 2.31

**Table 6:** Optimal objective function values for simulated annealing algorithm.

	Temperature	Waveform	Material	Frequency (Hz)	Peak
**Optimal conditions**	70 °C	sine wave	material 1	516,325.466	0.296
**Optimal objective function value**	0.007				

## Data Availability

The data used in this article comes from the dataset of question C in the 21st China Graduate Mathematical Modeling Competition of the “Huawei Cup” in 2024. The website is: https://cpipc.acge.org.cn/cw/hp/4 (accessed on 29 December 2024).

## Supplementary Materials

The following supporting information can be downloaded at: https://www.mdpi.com/article/10.3390/s26102919/s1, (1) Extract the attachment (data). The RAR file contains Attachment 1, which is the training set comprising four data tables representing measurements obtained from four different magnetic core materials (due to the complexity of these materials, we use Material 1, Material 2, Material 3, and Material 4 to denote them). The four data tables share the same structure. Column 1 contains temperature values: 25, 50, 70, and 90 °C; column 2 lists frequency values ranging from 50,000 to 500,000 Hz; column 3 shows magnetic core loss measured in watts per cubic meter; column 4 specifies excitation waveform types (sine, triangular, or trapezoidal); and columns 5 through 1029 present magnetic flux density data with 1024 equally spaced sampling points per cycle, measured in Teslas. Each row represents experimental results under a specific operating condition, with distinct rows corresponding to different operational scenarios. (2) Both Attachment 2 and Attachment 3 are test datasets. In Attachment 2: column 1 contains sample numbers; column 2 shows temperature; column 3 indicates frequency; column 4 lists core materials (Material 1, Material 2, Material 3, Material 4); and columns 5 through 1029 present magnetic flux density values (with identical structure, format, and meaning as those in Attachment 1). Attachment 3: column 1 contains the sample numbers; all other entries, except for the change from “magnetic core loss” to “magnetic core material,” maintain the same structure, format, and meaning as those in Attachment 1.

## Abbreviations

The following abbreviations are used in this manuscript:

XGBoost	eXtreme Gradient Boosting
MSE	Mean squared error
MAE	Mean absolute error
